# Impact of reduced frequency of phosphate testing on detected phosphate levels and phosphate prescription in critical care

**DOI:** 10.1186/cc13622

**Published:** 2014-03-17

**Authors:** D Hepburn, H Roberts, S Zouwail

**Affiliations:** 1 University Hospital of Wales, Cardiff, UK

## Introduction

Phosphate is essential for cell and bone function [1]. In critical illness, hypophosphatemia is common and practice is often to correct even mild derangement [2]. Its supplementation has significant cost and risk including hypotension and hypocalcaemia. We investigated whether changing frequency of routine serum phosphate testing had effects on the detected incidence of abnormal plasma levels and prescription of phosphate.

## Methods

This was a service improvement project using observational, anonymous data. We collected data on serum phosphate levels in a 33-bed ITU over two 6-month periods before and after introduction of a new testing regime (phases 1 and 2). In phase 1, phosphate levels were tested daily. In phase 2, phosphate levels were tested three times per week. Replacement was at clinical discretion. Pharmacy data on phosphate prescription were compared for both phases.

## Results

A total of 4,253 tests were performed in phase 1, and 3,641 in phase 2 - a reduction of 612 (14.4%). The ICU workload was similar in both phases. There was no significant difference in mean phosphate levels or detected incidence of abnormal phosphate levels in phase 1 versus phase 2. Mean level was 1.13 ± 0.40 mmol/l versus 1.14 ± 0.43 mmol/l (*P *= 0.42). Severe hypophosphatemia (<0.4 mmol/l) was relatively uncommon: *n *= 18 in phase 1 (0.42% of tests) versus 19 (0.52%), *P *= 0.42, in phase 2. Mild hypophosphatemia (0.4 to 0.7 mmol/l) was frequent, with 1,203 episodes (28.2% of tests) versus 1,088 (29.8%), *P *= 0.42. Hyperphosphatemia (>1.5 mmol/l) was also common in both phases with 608 detected episodes (14.3% of tests) versus 572 (15.7%), *P *= 0.49. Pharmacy data show phosphate replacement fell significantly, from 687 prescriptions in phase 1 to 395 in phase 2, with drug cost- savings estimated at £1,430.

## Conclusion

Reducing testing from daily to three times weekly was not associated with a significant change in mean phosphate levels nor with detection of abnormally high/low phosphate levels. Daily testing, however, is associated with higher rates of phosphate prescription. It is known that there is significant diurnal variation in serum phosphate [2] and we speculate that mild hypophosphataemia self-corrects without intervention. Treating mild hypophosphatemia may therefore not be indicated.
